# Effects of Marine Reserves versus Nursery Habitat Availability on Structure of Reef Fish Communities

**DOI:** 10.1371/journal.pone.0036906

**Published:** 2012-06-04

**Authors:** Ivan Nagelkerken, Monique G. G. Grol, Peter J. Mumby

**Affiliations:** 1 Department of Animal Ecology and Ecophysiology, Institute for Water and Wetland Research, Radboud University Nijmegen, Nijmegen, The Netherlands; 2 Southern Seas Ecology Laboratories, School of Earth and Environmental Sciences, The University of Adelaide, Adelaide, South Australia, Australia; 3 Marine Spatial Ecology Lab, School of Biological Sciences, University of Queensland, St Lucia Campus, Brisbane, Queensland, Australia; National Institute of Water & Atmospheric Research, New Zealand

## Abstract

No-take marine fishery reserves sustain commercial stocks by acting as buffers against overexploitation and enhancing fishery catches in adjacent areas through spillover. Likewise, nursery habitats such as mangroves enhance populations of some species in adjacent habitats. However, there is lack of understanding of the magnitude of stock enhancement and the effects on community structure when both protection from fishing and access to nurseries concurrently act as drivers of fish population dynamics. In this study we test the separate as well as interactive effects of marine reserves and nursery habitat proximity on structure and abundance of coral reef fish communities. Reserves had no effect on fish community composition, while proximity to nursery habitat only had a significant effect on community structure of species that use mangroves or seagrass beds as nurseries. In terms of reef fish biomass, proximity to nursery habitat by far outweighed (biomass 249% higher than that in areas with no nursery access) the effects of protection from fishing in reserves (biomass 21% lower than non-reserve areas) for small nursery fish (≤25 cm total length). For large-bodied individuals of nursery species (>25 cm total length), an additive effect was present for these two factors, although fish benefited more from fishing protection (203% higher biomass) than from proximity to nurseries (139% higher). The magnitude of elevated biomass for small fish on coral reefs due to proximity to nurseries was such that nursery habitats seem able to overrule the usually positive effects on fish biomass by reef reserves. As a result, conservation of nursery habitats gains importance and more consideration should be given to the ecological processes that occur along nursery-reef boundaries that connect neighboring ecosystems.

## Introduction

Coral reefs have important economic, biological and aesthetic values; they generate about $30 billion per year in fishing, tourism and coastal protection from storms [1]. However, they have seriously degraded in the last few decades through human and natural impacts, such as pollution, overexploitation, coral bleaching, coral diseases and hurricanes [2]. Of the island coral reef fisheries, 55% are currently unsustainable [3]. Overfishing is one of the principal threats to coral reef health and functioning, and has led to detrimental trophic cascades and phase shifts from coral reefs to macroalgal reefs at many Caribbean localities [4]–[6].

Marine Protected Areas (MPAs) are becoming an increasingly popular tool to protect reef biodiversity, support fisheries, and maintain ecological processes, albeit locally [7]. There are a wide variety of MPAs with different levels of protection, management approaches, and levels of allowable exploitation [8]. One of the key problems is that less than 1.4% of the world’s reefs lie inside no-take MPAs, while many MPAs are ‘paper parks’ which officially exist but lack sufficient compliance or effective enforcement against damage or exploitation by humans [9].

In theory, reserves may benefit fisheries through two, complementary mechanisms. First, by building up a stock of large-bodied, highly fecund fish, they protect a spawning stock that might help replenish stocks in exploited areas outside reserves [10]. Second, migration of adult fish outside reserve boundaries can support local fisheries, the so-called fishing the line [11]. The latter mechanism has been documented empirically for coral reefs [12] but empirical evidence for larval subsidy remains lacking, though would be expected in principle [13]. Nonetheless, for reserves to have any significant effect in a fisheries context, their first requirement is to establish an increase in fish biomass and/or change in fish community structure of focal species. However, protection from fishing is only one factor that affects the abundance of fish. The interactions between fisheries protection and other drivers of fish community structure are less well understood [14], [15].

A relatively poorly studied but very relevant concept for conservation biology, reserve design, and management of fisheries stocks is that of ecosystem connectivity. Inshore habitats such as mangroves and seagrass beds have long been considered to act as nurseries (i.e., juvenile habitats that contribute a higher than average biomass of individuals to the adult population compared to other juvenile habitats) for a suite of coral reef fish species (‘nursery species’), an assumption based on observations of high abundances of juvenile fish that these habitats typically harbor [16]–[19]. The nursery concept has been a paradigm for a long time due to lack of studies that showed actual emigration of fish from nurseries to adjacent reefs. Studies have recently provided compelling evidence showing that nursery habitats indeed replenish fish populations on directly adjacent reefs through ontogenetic, cross-ecosystem migrations [20]–[22].

While the nursery concept itself is not new, the quantification of its effects is still in a preliminary stage. There is lack of understanding of the exact degree to which nursery habitats subsidize reef populations and how far fish disperse from nurseries to replenish more distant reefs. Both marine reserves and nursery habitats may regulate fish abundance and community structure on coral reefs, however, we know little of their interactive effects. Coral reef reserves affect reef populations of nursery species differently than those of non-nursery species as they can protect the adult stages of the former from fishing, but potentially all demersal life stages of the latter. In contrast, protection of inshore nursery habitats would only affect the non-adult life stages of nursery fish, as fish migrate from nurseries to reefs as large juveniles or maturing fish [23]. So for species with a stage-structured life cycle whose adults and juveniles are spatially separated and utilize different types of ecosystems, what are the benefits of protecting juvenile nursery grounds in combination with maintaining nursery-reef connectivity versus protecting the adult reef habitat near nurseries? Are they both important, and if so, are they equally important, or is there perhaps a synergistic effect on reef fish populations when reef reserves occur near nurseries? Considerations like this are important for managing reef fish populations, yet empirical data are needed for an understanding of such processes.

Here, we study a series of marine reserves in the Cayman Islands (Caribbean Sea) which vary in their proximity to mangrove nurseries. We evaluate the relative effects of reserve implementation and mangrove/seagrass nursery function on the fish community structure and biomass of fish species on coral reefs.

## Materials and Methods

### Study Area

**Figure 1:**
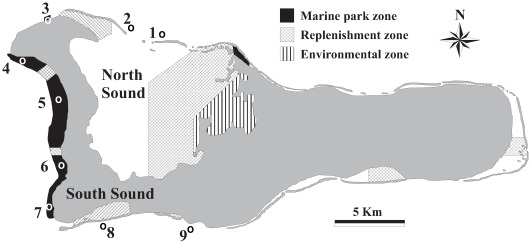
Map of the study area. Numbers 1–9 show the sampled reef sites (O) in fishery reserves (marine park zone) and in fished areas (non-reserves). Reef sites close (<1 km distance) to nursery habitats are site # 1, 2, 3, and 7, while those isolated (>3.5 km distance) from nurseries are site # 4, 5, 6, 8, and 9; site # 3 falls completely within the small northern marine park zone. The position of the replenishment zones (line fishing allowed on fish>20 cm in body length) and the environmental zone (no fishing of any kind allowed) is also indicated. Location of the various zones was obtained from the Cayman Islands marine park brochure. Grey represents land mass.

The study was executed on the Caribbean island of Grand Cayman (Cayman Islands). The island has a continuous fringing reef that surrounds the island. The shelf is relatively narrow (300–900 m) and turns into a steep submarine wall at a depth of>20 m. Mean (±SD) live benthic cover (stony corals, soft corals, sponges, etc.) on the reefs studied was 42±15%, while reef elevation above the substratum was 0.9±0.2 m. Several marine fishery reserves (‘marine park zones’) exist on the island, which have been largely protected against fishing since 1986 (Fig. 1). In the marine reserves, anchoring and extraction of dead or living marine life is not permitted, except anchoring in sandy areas and line fishing from shore and beyond the reef wall at depths>25 m (minimum size limit 20 cm fish length). Fishing pressure on the island is relatively low compared to other Caribbean islands and reef fishery resources are not overexploited [3], [24].

The island harbors one very large (North Sound) and various small lagoons (Fig. 1). The substratum of all lagoons is dominated by seagrass cover (*Thalassia testudinum*), but only the North Sound harbors inundated mangroves (*Rhizophora mangle*) along most of its shoreline. Seagrass beds do not occur outside of the island’s lagoons. A large portion of the lagoons consists of ‘replenishment zones’ (Fig. 1) where line fishing is allowed anywhere within the zone (minimum size limit 20 cm), but the use of spears, traps, nets, and fish poison is prohibited; two similar replenishment zones are found on the reef at the western side of the island. The North Sound also has an ‘environmental zone’ where anchoring, in-water activities, and any extraction of dead or living marine life are prohibited. In all other undesignated areas of the island, fishing is allowed but permits are required for spearfishing and use of fish traps, while the minimum size limit of fish remains 20 cm.

### Sampling Design

Marine fishery reserves were selected that were either close to (<1 km; reef sites # 3 and 7) or isolated (3.5–10 km; reef sites # 4, 5 and 6) from mangrove/seagrass habitats (further referred to as ‘nurseries’) in lagoons (Fig. 1). The same selection was made for fished areas (non-reserves): close to (reef sites # 1 and 2) or isolated (reef sites # 8 and 9) from nurseries. All selected reserve and non-reserve sites, except sites # 1, 2, and 8 were accessible from shore. The two reef replenishment zones on the western side of the island (see Fig. 1) were not considered to function as nurseries for reef fish as line fishing is allowed anywhere on these reefs (also from boats), while the 20 cm size limit in these zones is also applicable to other reef areas. Therefore, all reserve sites are considered as independent reef sites that were selected from a continuous reef along the coastline of the island. Nagelkerken et al. [25] identified 17 reef fish species that show variable degrees of dependence on nurseries during the juvenile life stage (further referred to as ‘nursery species’). All nursery species and their congeners were selected in the present study, viz. all species belonging to the families of Acanthuridae, Chaetodontidae, Gerreidae, Haemulidae, Lutjanidae, Scaridae, Sphyraenidae, and in addition species of Mullidae as juvenile *Mulloidichthys martinicus* are sometimes found in mangroves [26]. In total, 30 highly common species were included in the surveys.

Using a stationary point count visual census technique [27] the number of individuals for each species and their total body length (TL to the nearest cm) were quantified at each of the nine sites in reef quadrats of 10×10 m at depths of 6–15 m. Depths>15 m were not sampled as preliminary surveys showed low abundances of nursery species on the steep reef walls of the island. Per site, 12 replicate quadrats were surveyed for 10 min each. The first 7 min of a survey was used to quantify mobile fish, while the last 3 min were used to count site-attached fishes. Studies have shown that once-only visual surveys of protected versus fished areas provide comparable results as long-term monitoring with respect to fish biomass distribution [28].

### Statistical Analysis

For each fish counted, total body length (TL) was transformed to weight (W) using the equation W = a×TL^b^, with species-specific values for *a* and *b* obtained from Bohnsack and Harper [29]. Biomass was used instead of densities as it is a better measure of productivity. Per species, fish biomass was averaged across quadrats at each site. Bray-Curtis similarity coefficients were calculated among sites using untransformed mean biomass per species. The similarity matrix was used to generate a non-metric multi-dimensional scaling plot. The importance of fishery reserve (present vs. absent) and nursery proximity (close vs. isolated) was tested using a 2-way ANOSIM with replication [30]. SIMPER analysis was then used to identify the species responsible for any significant patterns [30].

More detailed analysis of reserve and nursery effects was done by size spectrum analysis. For each species, biomass per 5-cm length classes was first summed per reef site. Size spectra were then plotted for all common large-bodied species (i.e., species that can attain sizes >25 cm TL) using only isolated reserve and fished sites (to avoid confounding effects of nursery proximity); at 25 cm TL, clear differences in biomass were present between reserves and fished areas for most of the species. This 25-cm cut-off level based on underwater visual estimations is nearly equal to the 20-cm minimum size limit for landed fishes (due to enlargement of an object seen through a dive mask). For the analysis, biomass of species was pooled for all fishes≤25 cm and>25 cm TL, respectively, based on data from all surveyed reef sites. The effect of reserve presence and nursery proximity was then tested using linear mixed-effects models on log-transformed data, with reserve presence and nursery proximity as crossed fixed effects and site as a random factor.

## Results

**Figure 2:**
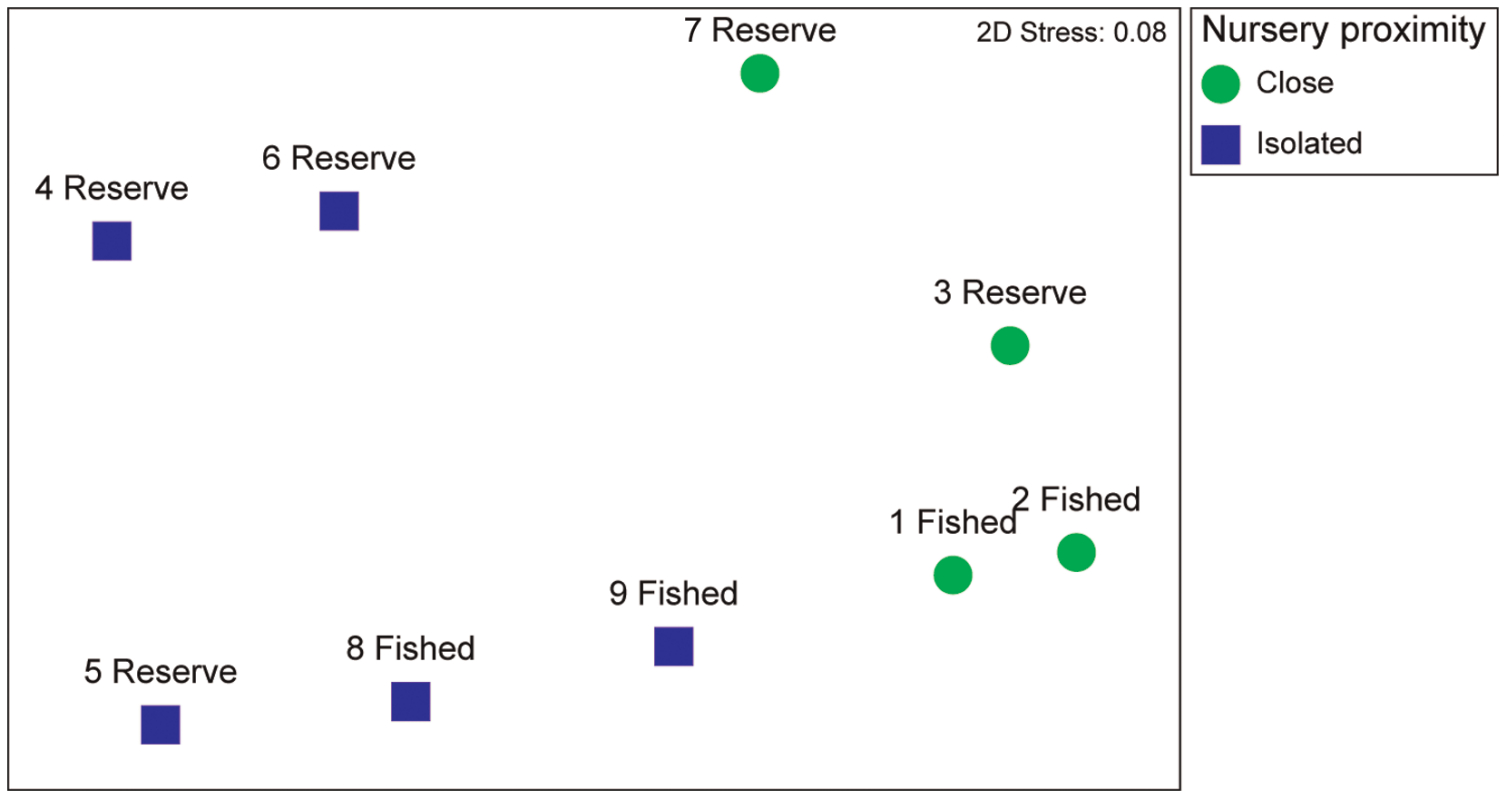
Non-metric multi-dimensional scaling plot for biomass of nursery species. The plot shows the ordination of the fish community at reef sites (numbered 1–9, see Fig. 1) that differ in fishery protection (reserve vs. fished) and nursery proximity (close vs. isolated).

**Figure 3:**
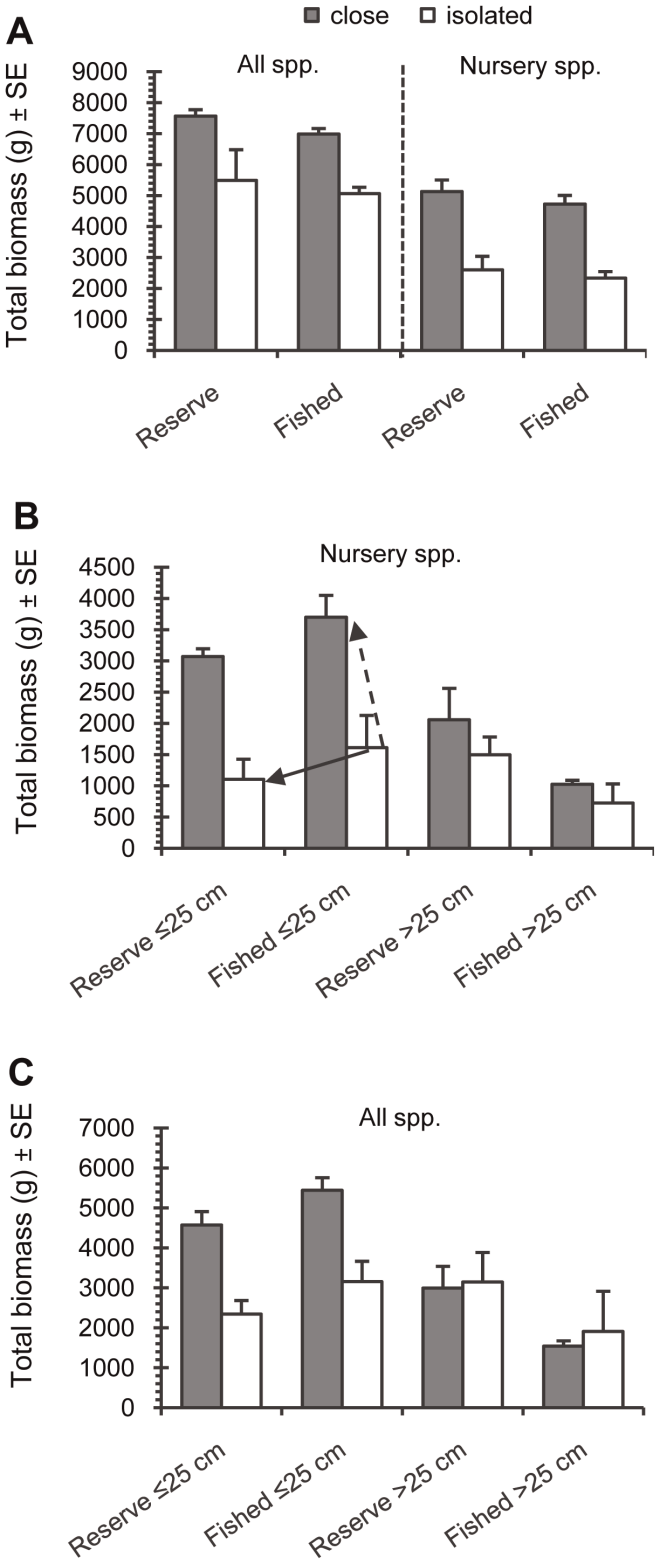
Fish biomass in marine reserves vs. fished areas with different proximity to nurseries (close vs. isolated). Mean total biomass per 100 m^2^ (±standard error) across reef sites is shown for the entire size range (A) of nursery species and all species, and split (B, C) for small (≤25 cm total length) and large (>25 cm total length) fish. The black arrow indicates the reserve effect in absence of nurseries, whereas the dashed arrow indicates the nursery habitat effect in fished areas on small individuals of nursery species.

For the structure of the entire fish assemblage, nursery habitat proximity was not significant (p = 0.07), but had a moderately strong effect (Rho) of 0.5, whereas reserve presence had no significant effect and a low R value (2-way ANOSIM, R = 0.19, p = 0.33). For reef fish that use mangrove/seagrass nurseries as juveniles (nursery species), the effects were much stronger such that nursery proximity had a very strong effect on their structure (Fig. 2; R = 0.94, p = 0.03) and total biomass (see Fig. 3b), whereas reserve presence had no overall effect on community structure (R = 0.46, p = 0.13). Even though the reef sites were located at different parts of the island, sites close to nurseries were more similar to one another in their community structure than to the isolated sites (Fig. 2). In decreasing order of importance, *Haemulon flavolineatum*, *Lutjanus apodus*, *L. analis*, *H. sciurus*, *L. mahogoni*, *Ocyurus chrysurus*, *H. plumierii*, *Scarus iseri*, and *S. guacamaia* contributed most (SIMPER analysis, cumulative contribution: 91%) to the differences in assemblage structure (n = 17 spp.), with their biomass being higher at sites close to vs. isolated from nurseries, except *L. analis* which showed the opposite pattern (Table 1). Considering species presence/absence alone, nursery species were observed at reserve as well as fished reef sites, and at sites close to nurseries as well as on isolated reefs.

**Table 1:** Results of SIMPER analysis for nursery species, showing which species best explained differences in fish community between sites close to vs. isolated from nurseries (average dissimilarity = 53.2).

Species	Biomass (g)		Average dissimilarity	Contribution (%)	Cumulative contribution (%)
	Nursery: close	Nursery: isolated			
*Haemulon flavolineatum* (French grunt)	886	214	9.2	17.3	17.3
*Lutjanus apodus* (schoolmaster snapper)	989	426	7.4	13.9	31.1
*Lutjanus analis* (mutton snapper)	320	618	6.6	12.4	43.5
*Haemulon sciurus* (bluestriped grunt)	618	204	5.6	10.5	54.0
*Lutjanus mahogoni* (mahogony snapper)	354	191	5.2	9.7	63.7
*Ocyurus chrysurus* (yellowtail snapper)	345	17	4.8	9.0	72.7
*Haemulon plumierii* (white grunt)	435	91	4.6	8.6	81.3
*Scarus iseri* (striped parrotfish)	492	315	3.0	5.6	86.9
*Scarus guacamaia* (rainbow parrotfish)	169	9	2.3	4.3	91.2

When fish biomass was analyzed irrespective of body size, biomass of species that use a mangrove/seagrass nurseries as well as all species was significantly higher at sites close to nurseries than at isolated reef sites, independent of reserve effect (Fig. 3a; linear mixed effect models t_nursery_ = 2.6 for nursery species and t_nursery_ = 2.9 for all species; t_reserve_<0.3 in both cases). No reserve effect was noticeable, however, for either reef sites close to or sites isolated from nurseries (t_reserve_<0.3 in both cases in linear mixed effects models). Size spectrum analysis showed that the response of large-bodied individuals (>25 cm TL) to protection from fishing in reserves and nursery access depended on whether they used nurseries as juveniles. For those species that used nurseries, total biomass was significantly greater in reserves (compared to fished areas) and when nursery access was high (vs. nursery-isolated areas) (Table 2; Fig. 3b). However, when the analysis was performed for all species, the total biomass was only affected by reserve status (Fig. 3c). A different pattern emerged for smaller-bodied fishes (≤25 cm TL). The abundance of species that utilized nurseries was positively associated with the presence of nurseries (Fig. 3b). However, their collective biomass was significantly lower in reserve areas than in fished areas (Table 2 ); a similar pattern was observed for the relatively small-bodied species *Haemulon flavolineatum*, *H. plumierii*, *Chaetodon capistratus*, and *Acanthurus chirurgus* which were only observed as individuals of≤25 cm TL. When the analysis was extended to include nursery as well as non-nursery species, the nursery impact remained but no effect of reserve presence was detected (Fig. 3c, Table 2).

**Table 2:** Results of linear mixed-effects models for fish biomass of nursery as well as all species with reserve presence and isolation from nurseries as crossed fixed effects and site as a random factor.

Comparison	Factor	Estimate (SE)	t value	
Nursery spp. ≤25 cm	intercept	7.94 (0.16)	50.86	
	reserve effect	−0.40 (0.17)	−2.35	*
	nursery isolation	−0.86 (0.16)	−5.36	*
Nursery spp. >25 cm	intercept	4.89 (0.61)	7.97	
	reserve effect	1.82 (0.64)	2.85	*
	nursery isolation	−1.25 (0.60)	−2.10	*
All spp. ≤25 cm	intercept	5426.3 (463.2)	11.72	
	reserve effect	−838.1 (517.9)	−1.62	NS
	nursery isolation	−2254.8 (517.9)	−4.35	*
All spp. >25 cm	intercept	6.13 (0.48)	12.66	
	reserve effect	0.98 (0.49)	1.99	*
	nursery isolation	0.15 (0.45)	0.34	NS

No. of observations = 108,

* P<0.05, NS = not significant.

Size indicates total fish length.

## Discussion

Proximity to mangrove/seagrass nursery habitats by far outweighed the effects of protection from fishing (i.e., reserve effect) for reef fish that use mangrove/seagrass nurseries and whose body length was less than 25 cm. Whereas reserves had on average 21% lower biomass of small fish compared to fished areas (when combining both nursery treatments), presence of nursery habitat biomass led to a 249% higher biomass compared to reefs without nearby nursery habitat access (combining both protection treatments). The lower biomass of small fishes in reserves is not surprising, as the protection of large fish can result in higher predation rates on prey fish compared to fished areas [31], [32]. Indeed, biomass of several nursery species that are predators in their adult stage was higher for individuals >25 cm TL in reserves (e.g., *Lutjanus apodus*, *L. mahogoni*, *Ocyurus chrysurus*) which may partly account for the lower abundance of potential prey fish in fished areas. Irrespective of fishing, nursery species were more abundant on reefs with nursery access than on nursery-isolated reefs due to the relatively short distances that these fish disperse [33]–[36]. The present study indicates that the magnitude of this effect is such that fished areas with nursery access can have much higher standing stocks (in this case 2.5 fold) of small-bodied fishes than marine reserves that do not have nursery access.

For large-bodied nursery fish (>25 cm TL), the magnitude of nursery effect was more subtle, with reserve effect being greater than nursery effect. Combining the two treatments, biomass in reserves was on average 203% of that in fished areas, while mean biomass in areas close to nurseries was only 139% of that in nursery-isolated areas. In fact, nursery-isolated reserves showed higher biomass of large nursery species than fished areas close to nurseries. This difference compared to smaller fish can be explained by large individuals dispersing farther away, e.g. [33] from nursery areas and being more heavily targeted. Compared to fished nursery-isolated areas (mean biomass = 0.7 kg per 100 m^2^), biomass was 1.4 times higher in fished areas near nurseries, 2.1 times higher in reserves isolated from nurseries, and 2.8 times higher in reserves near nurseries. This indicates that nursery presence and protection from fishing in reserves had an additive effect on the reef biomass of large nursery fish, with reserve presence contributing to a higher degree than nursery presence. Protection of the larger individuals of nursery species should thus not be restricted to areas close to nurseries, although they benefited most from fishery protection near nurseries. Nevertheless, nursery-access enhanced biomass of large nursery species in fished as well as reserve areas.

The species that showed the largest contribution to differences in nursery fish community structure between areas close to and isolated from nurseries all belonged to the families of grunts, snappers, and parrotfishes. These families all form an important component of Caribbean line and trap reef fisheries [37]. In addition, some provide important ecological roles in terms of reef functioning. Large-bodied snappers such as *Lutjanus apodus* and *Ocyurus chrysurus* form part of the suite of reef fish predators that can exert a top-down effect on the structure of marine communities, e.g. [38], [39]. Small fishes like some species of grunts act as prey species supporting piscivore populations, e.g. [40], [41], especially the common and small-bodied French grunt *Haemulon flavolineatum*. Parrotfish take up an important ecological role as grazers protecting coral reefs from algal overgrowth [5], [42] and one of the most abundant Caribbean parrotfish is the nursery species *Scarus iseri* [25], [43]–[45]. Biomass (entire size range) of all of the above species was higher on reefs close to than far away from nurseries, which underlines the importance of ecosystem connectivity for reef resilience and ecosystem functioning [46].

Healthy nursery habitats may show an overarching effect on populations of some reef fish species compared to marine reserves. About half of the lagoon of North Sound consists of seagrass beds where only line fishing is allowed and only on individuals of>20 cm body length, thus sparing juvenile fish and the source area of new recruits. In addition, a significant area of mangrove and seagrass habitat in the lagoon has been appointed as a no-take zone (‘environmental zone’). Although we do not have data from different lagoons to compare productivity of nursery habitats, the data at least show that nursery habitats which receive a certain level of protection can be highly productive for sub-adult fishes, to a degree that the reef-ward flow of this productivity (i.e., fish movement) overrules the usually positive effects on fish biomass of reef reserves, e.g. [10]. The magnitude of this effect was such that maintenance of health and productivity of nursery habitats should receive more weight than perhaps considered previously. In this light, management efforts and scientific studies should also focus in greater detail on nursery-reef boundary areas, as these form important ecological corridors that maintain connectivity. As worldwide, MPAs most often include coral reefs (about 980 [9]) as opposed to mangrove (roughly 237 [47]) or seagrass nurseries (about 247 [48]) more efforts are needed to establish reserves that specifically consider nursery species. This is especially true for the Caribbean where strong nursery–reef connectivity exists [19]. In the Indo-Pacific region, the seascape structure is often different as extensive mangrove systems occur that are located at greater distances from coral reefs compared to Caribbean islands. Moreover, higher tidal ranges in much of the Pacific often make mangrove nurseries an ephemeral habitat whereas most mangroves are permanently inundated in the Caribbean. The importance of ontogenetic connectivity may be lower in parts of the Indo-Pacific leading to different interactive effects with reef reserves. However, a number of Indo-Pacific studies have indicated significant impacts of mangrove isolation or absence on coral reef fish abundance [27], [49], [50], indicating that for a number of Indo-Pacific locations the current findings may applicable too. Some potential support for this is provided by a very recent study showing increased fish densities in reserves close to mangroves [51], but in that study both habitats were located within the boundaries of a nursery bay, likely restricting the conclusions to recurring tidal connectivity instead of permanent life cycle connectivity. A systematic analysis of mangrove and reserve effects along a gradient of tidal range and biodiversity would be useful for the Indo-Pacific region.

### Conclusions

The relative importance of nursery habitat and marine reserve presence on coral reef fish community structure depends on fish size and whether fish use mangrove/seagrass nurseries. Large individuals of nursery species which are commercially exploited seem similarly susceptible to fishing as other species and benefit most from protection in areas close to nurseries. For small individuals of nursery species, nursery habitat presence by far outweighed the effects of protection from fishing in marine reserves. The present study shows how ecosystem connectivity adds an additional level of complexity to marine reserve design and functioning.
